# Supplementary material for: Effects of Mustelid gammaherpesvirus 1 (MusGHV-1) Reactivation in European Badger (Meles meles) Genital Tracts on Reproductive Fitness
Source: Pathogens. 2020 Sep 20;9(9):769. doi: 10.3390/pathogens9090769 (PMC7559395; doi:10.3390/pathogens9090769)
Supplement: Supplementary file 1 [file pathogens-09-00769-s001.zip › pathogens-905329 - supplementary/pathogens-905329 - supplementary S3 (revised).docx]

List of badger samples for histology (collected by Ming-shan Tsai at Feburary 2020)

| 19PB3066 Female, was pregnant, vaginal tract |
| --- |
| 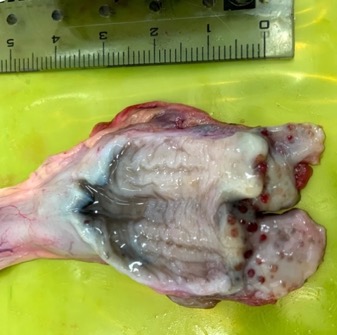Histopathological examination showed epithelial hyperplasia with a subepithelial chronic inflammatory infiltrate of macrophages, lymphocytes, plasma cells and a few eosinophils. |
| 20PB0384 Female, RP?L, vaginal tract |
| Histopathological examination showed epithelial hyperplasia. There was a low grade diffuse subepithelial chronic inflammatory infiltrate primarily lymphocytes with occasional perivascular foci. 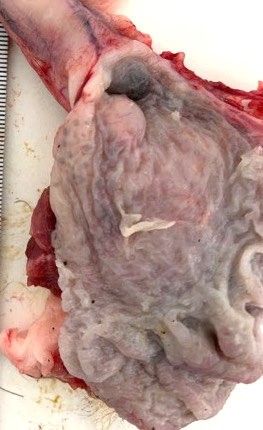 |
| 20PB0398 Female, Pregnant (2p), vaginal tract |
| 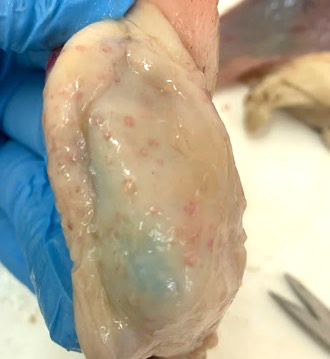Two tissues examined: Histopathological examination showed tissue oedema with diffuse and focal subepithelial inflammatory cells primarily macrophages, plasma cells, lymphocytes and eosinophils. The eosinophils were especially prominent in the deeper layers. In the second sample there was epithelial hyperplasia, a folded surface epithelium with subepithelial inflammatory foci of macrophages, lymphocytes, plasma cells and eosinophils. |
| 20PB0444 Female, NP, cervix |
| 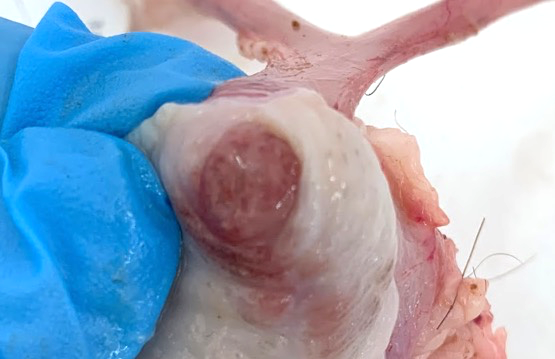Histopathological examination showed a diffuse low grade chronic inflammatory infiltrate. |
| 20PB0527 Female, vaginal tract |
| 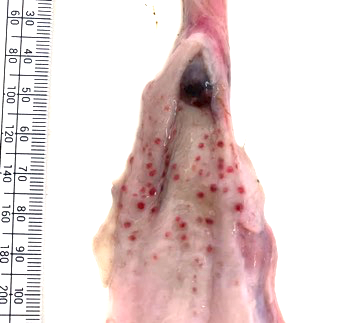Histopathological examination showed a diffuse subepithelial chronic inflammatory infiltrate. Occasional subepithelial focal accumulations of cells were primarily macrophages, lymphocytes, plasma cells with an occasional neutrophil. |
| 20PB0350 Male penis |
| Histopathological examination showed epithelial hyperplasia with keratinisation of superficial cells. There were subepithelial foci of chronic inflammatory cells, primarily lymphocytes.  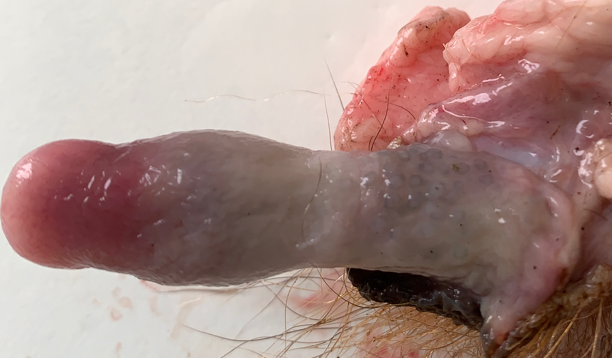 |
| 20PB0496 Male penis |
| 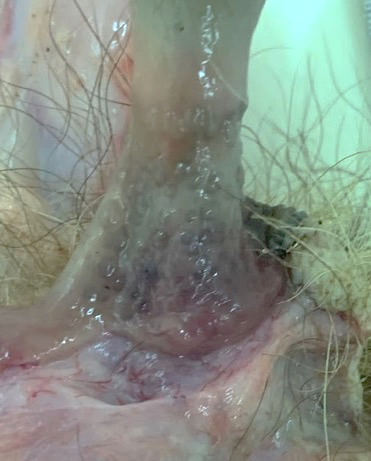Histopathological examination showed epithelial hyperplasia with intranuclear inclusions and there was a subepithelial inflammatory infiltrate of macrophages, lymphocytes and plasma cells. |
